# Application of ultrasound elastography for monitoring the effects of TβR1 shRNA therapy on hepatic fibrosis in a rat model

**DOI:** 10.1371/journal.pone.0253150

**Published:** 2021-06-28

**Authors:** Xiangzhou Shi, Jinghua Li, Binying Min, Ruijing Yang, Chunxiang He, Yilin Yang

**Affiliations:** Department of Ultrasound Diagnosis, Tangdu Hospital, Fourth Military Medical University, Xi’an, China; Fudan University, CHINA

## Abstract

**Background:**

To investigate the application of ultrasound elastography in monitoring the effects of the transforming growth factor (TGF)-β1 signaling pathway-targeted combination therapy for hepatic fibrosis.

**Methods:**

1. Short hairpin RNA (shRNA) constructs targeted towards *TβR1* were designed, synthesized, and packaged using an adeno-associated virus (AAV), and the effective target shRNA was selected based on transfection results. 2. Fifty rats were randomly allocated (n = 10 per group) to the (A) control group, (B) model group, (C) 0-week therapy group, (D) 4-week therapy group, and (E) combination therapy group. At weeks 2, 4, 6, 8, 10, and 12, acoustic radiation force impulse (ARFI) elastography was used to measure the liver stiffness, inner diameter of the portal vein diameter, and blood velocity; radio frequency ultrasound imaging was used to measure the abdominal aortic elasticity parameter and pulse wave velocity (PWV) of the rats. 3. At week 12, portal vein puncture was performed to measure the portal venous pressure, and rat liver specimens were obtained for the pathological measurement of the degree of hepatic fibrosis.

**Results:**

1. An shRNA interference sequence targeted towards *TβR1* was successfully designed, screened, and packaged using an AAV, and small-animal imaging results indicated expression of the specific shRNA in the liver. 2. At week 12, the ultrasound elastography results were significantly different between the experimental groups and the control group (p < 0.01); among the experimental groups, differences were significant between the therapy groups and the model group (p < 0.01). For groups C and E, the therapeutic effects on hepatic fibrosis in rats were significant, with the pathological results indicating a significant reduction in the degree of hepatic fibrosis (p < 0.01). The therapeutic effectiveness of group D was less than that of group C (p < 0.05). Significant differences existed between the portal venous pressure of the experimental groups and of the control group (p < 0.01). For the abdominal aortic elasticity parameter measured by radio frequency ultrasound imaging, differences existed between the values obtained from the experimental groups and from that of the control group (p < 0.05), while statistically significant differences were not found among the various experimental groups. 3. Continuous ultrasound examination results indicated that the elasticity value of group A was significantly different from those of the other groups after 2 weeks of model establishment (p < 0.01); after 6 weeks, the elasticity values of groups C and E were significantly different compared with those of groups B and D (p < 0.01). For the abdominal aortic elasticity parameter and pulse wave velocity (PWV), there were no significant differences among the various groups (p > 0.05).

**Conclusion:**

CCl4-induced hepatic fibrosis can be treated through shRNA silencing of TβR1. Ultrasound ARFI elastography is superior to external force-assisted elastography as it can reflect the degree of fibrosis in moderate to severe hepatic fibrosis and the variations in the degree of fibrosis after treatment. Portal venous pressure was positively correlated with the degree of fibrosis; with early combination therapy, both the degree of fibrosis and portal venous pressure could be effectively reduced.

## Introduction

One of the key pathological features of hepatic fibrosis is that the imbalance of the extracellular matrix (ECM). Studies [1,2] have shown that early treatment of this imbalance can prevent or even reverse the progression of fibrosis. Transforming growth factor (TGF)-β1 has been identified by existing research as the most important fibrosis-mediating cytokine; therefore, the pathological process of hepatic fibrosis may be mitigated by interfering with TGF-β1 activity [3,4]. In addition, the TGF-β1 can stimulate the production of endothelin (ET), and endothelin can play a role in the course of liver disease through the receptor; therefore, the ET receptor can be a target for treating hepatic fibrosis [5]. Liver biopsy has long been the gold standard for diagnosis of hepatic fibrosis. However, its invasive nature and the constraints in specimen collection limit its applications, especially in observing the effects of anti-fibrosis therapies [6,7]. Therefore, a non-invasive examination method would be a significant tool for monitoring disease progression and the effects of therapy in hepatic fibrosis patients.

Ultrasound elastography is a recently developed technique that utilizes shear waves to reflect changes in an elastic medium. It can be used to monitor the extent of liver hardening that occurs during hepatic fibrosis due to the deposition of ECM. Indeed, several studies have reported different elasticity values when ultrasound elasticity imaging was performed on chronic liver disease patients with varying degrees of hepatic fibrosis [8–11]. However, further investigation is required to ascertain if this technique can reflect the changes in liver stiffness as an indicator of liver pathology after anti-fibrosis therapy. This study aimed to utilize ultrasound elastography imaging to measure the changes in liver stiffness after TGF-β1 signaling pathway-targeted combination therapy for hepatic fibrosis so as to determine if this technique can be used as a non-invasive method for monitoring the effects of anti-fibrosis therapy.

## Materials and methods

### 1. Care and maintenance of the experimental animals

A total of 50 male Sprague Dawley (SD) rats weighing 200±20g (age, 8weeks) were used in the experiment purchased from the Experimental Animal Center of Fourth Military Medical University. This study was approved by the Ethics Committee for Animal Experiments in the Fourth Military Medical University. All applicable international, national, and/or institutional guidelines for the care and use of animals were followed. Animals were given ad libitum access to water and food during the experiment and housed in an environment at 24±2°C with the humidity of 50%±10% and 2 h/12 h light-dark cycle. In the process of feeding rats, we mainly measure the weight, water consumption, food consumption and body temperature of rats to determine their health status. They were housed for 7 days and then randomly divided into five groups.

### 2. Experimental materials

TRIzol, RNase-free DNase I and reverse transcription kits were purchased from Promega Corporation (USA). Quantitative fluorescence PCR (QF-PCR) kits were purchased from Applied Biosystems (USA). QF-PCR primers were synthesized by Beijing SBS Genetech (China). Mouse anti-rat monoclonal antibodies against collagen I, collagen III, matrix metalloproteinase 1 (MMP1), tissue inhibitor matrix metalloproteinase 1 (TIMP1), TGF-β1, and transforming growth factor-β type I receptor (TβR1) were purchased from R&D Systems (USA). Mouse anti-rat β-actin monoclonal antibody was purchased from Sigma-Aldrich (USA).

### 3. Experimental methods

#### 3.1 Construction of short hairpin RNA (shRNA) interference vector against rat TβR1

Candidate target shRNA sequences were designed based on the sequence of the rat *TβR1* using the online selection tool by Life Technologies (USA). The rat *TβR1*-targeted recombinant adeno-associated viruses (AAV8-TβR1) were packaged and synthesized by Shanghai GenePharma Co., Ltd. (China), and the effective recombinant AAV8- TβR1 virus was screened based on the results of *in vitro* transfection with the AAV8 vectors. The selected vector was stored in a −80°C freezer at a titer of 1 × 10^13^ viral genomes (v.g.) per milliliter and diluted 10-fold to a titer of 1×10^12^ v.g./ml (working concentration) before use.

#### 3.2 Establishment of animal model and grouping of experimental animals

**Fig 1:**
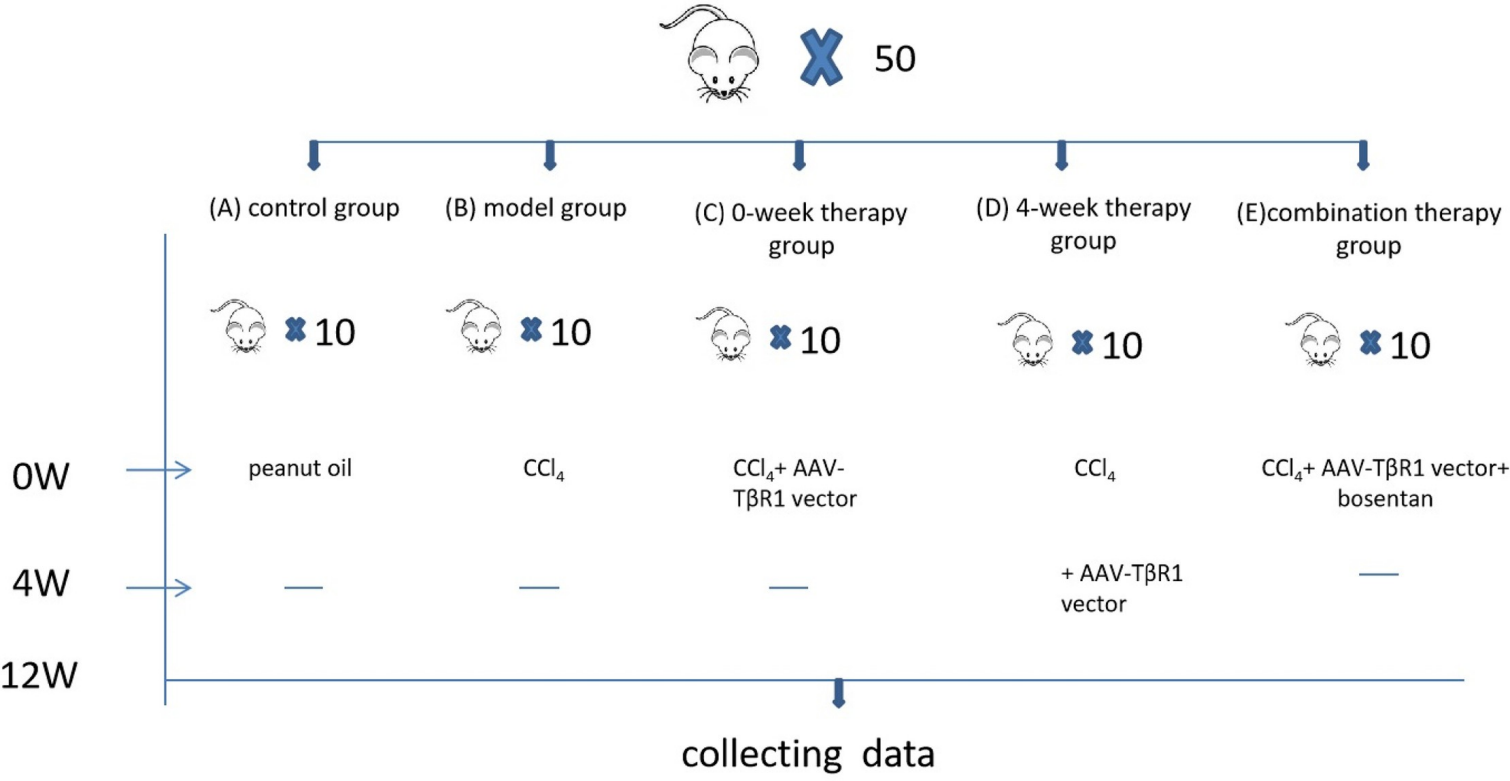
Experimental grouping diagram.

Fifty SD rats were randomly allocated to 5 groups (n = 10 per group): (A) control group, (B) carbon tetrachloride (CCl_4_) model group, (C) 0-week therapy group (CCl_4_ + AAV8- TβR1 0W), (D) 4-week therapy group (CCl_4_ + AAV8- TβR1 4W), and (E) combination therapy group (CCl_4_ + AAV8 -TβR1 + bosentan) (Fig 1). For the control group, intraperitoneal injections of peanut oil (0.3 ml/100g body weight) were administered twice weekly; for the other groups, intraperitoneal injections of 40% CCl_4_ (0.3 ml/100 g body weight) were administered twice every week until 12 weeks. For the 0-week therapy group, the selected AAV8-TβR1 vector (250 μl of 1 × 10^12^ v.g./ml) was administered via tail vein injection immediately after the start of model establishment. For the 4-week therapy group, tail vein injections of the selected vector (250 μl of 1 × 10^12^ v.g./ml) were administered 4 weeks after the start of model establishment. For the combination therapy group, the selected vector (250 μl of 1 × 10^12^ v.g./ml) was administered via tail vein injection immediately after the start of model establishment. For the combination therapy group, the selected vector (250 μl of 1 × 10^12^ v.g./ml) was administered via tail vein injection immediately after the start of model establishment; this was followed by once-daily intragastric administrations of the ET-1 antagonist, bosentan (200 mg/kg body weight). For the model group, the recombinant virus was replaced by an equivalent volume of sterile saline. During the experiment, we strictly abide by the laboratory operation specifications, especially the laboratory operation and technical procedures related to virus transfection, and there are no unanticipated adverse effects that took place during the study.

#### 3.3 Ultrasound measurements of parameters

**Fig 2:**
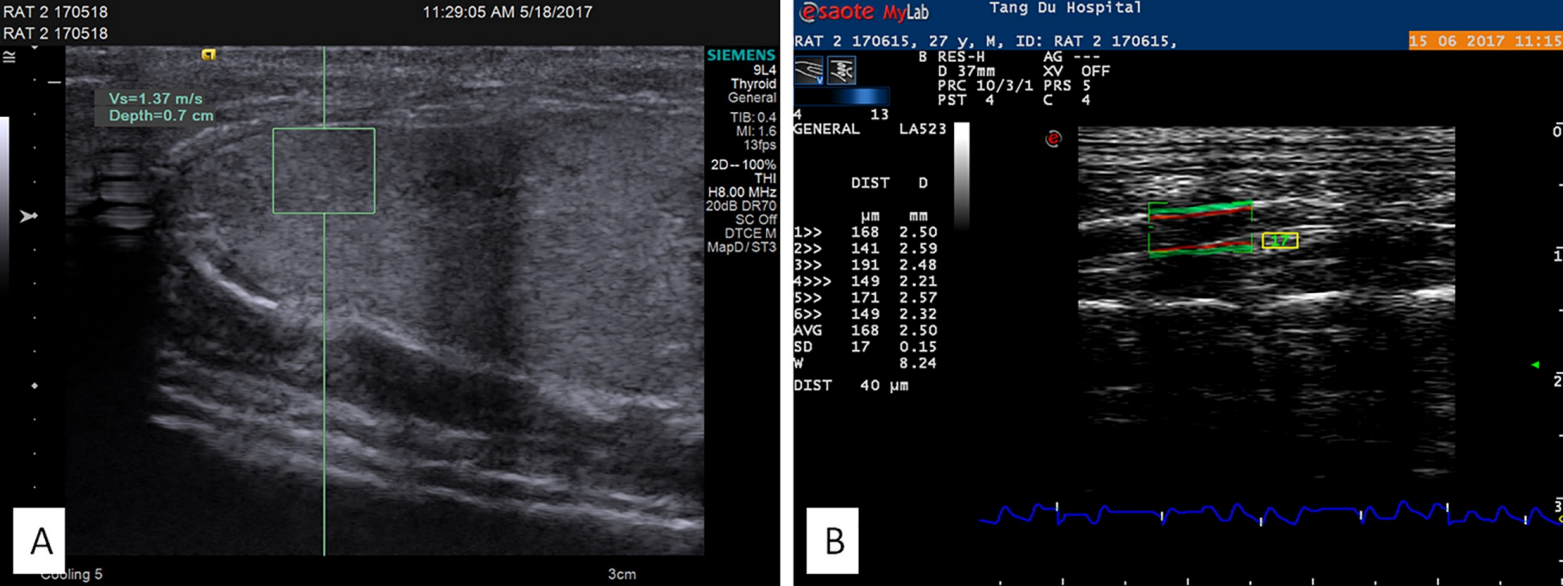
A. ARFI elastography was performed to measure the rat liver stiffness; B. Radio frequency ultrasound imaging was performed to measure the abdominal aortic elasticity parameter and the pulse wave velocity (PWV) of the rats.

At weeks 2, 4, 6, 8, 10, and 12 after model establishment, ultrasound measurements of parameters were performed as follows: (1) Using the linear array 9L4 probe (frequency range: 7–9 MHz) of a color Doppler ultrasound system VC30D (Siemens ACUSON S2000, USA, Serial No. 212448), acoustic radiation force impulse (ARFI) elastography was performed to measure the rat liver stiffness. Prior to the examination, rats were placed with Isoflurane inhalation anesthesia to induce sleep and anesthesia (as preliminarily ascertained). Te glass jar was securely covered for 2 min. each rat was anesthetized and fixed on a bench, and body hair removal was performed on the region to be examined. During the ARFI imaging process, a real-time two-dimensional image of the liver was displayed. A region of interest (ROI) (5 mm × 10 mm) was selected from clear ultrasound images using the ROI cursor. Areas with large blood vessels and bile ducts were avoided, and images of the liver parenchyma (at a fixed depth of 0.7 cm below the liver capsule) were acquired. Upon detection of uniform echoing within the frame, the probe button was pressed to freeze the image and display the depth and shear wave velocity (m/s) of the ROI in the system. Ten separate values were obtained through repeated measurements, and the median value was recorded as the final result [12]. The portal vein diameter and blood velocity were measured at the portal area (Fig 2A). (2) Using a linear array LA523 probe (frequency range: 4–13 MHz) of an Esaote MyLab 60 system V11.1 (Esaote, Italy, Serial No.3846), radio frequency ultrasound imaging was performed to measure the abdominal aortic elasticity parameter and the pulse wave velocity (PWV) of the rats. The ultrasound system was equipped with the RF-data technology and Mylab Desk image analysis software (Esaote, Italy). The frame of the ROI was shifted to the retrohepatic abdominal aortic region for measurements, and the system automatically recorded the intima-media thickness (IMT) and elasticity parameters of 6 cardiac cycles. When the standard deviation of the IMT < 30, the indicator turned green, suggesting that the measurements were stable, and the values of IMT; PWV; arterial wall dilation coefficient (DC); arterial wall compliance coefficient (CC); sclerosis indices α and β; and augmentation index (Aix) were automatically exported as the final results (Fig 2B) [13].

#### 3.4 Small animal imaging

For the 0-week and 4-week therapy groups, the selected AAV8 -TβR1 (250 μl, titer: 1 × 10^12^ v.g./ml) was administered via tail vein injections; for the control and model groups, an equivalent volume of saline (250 μl) was administered via tail vein injections. At weeks 2, 4, 6, 8, 10, and 12 after model establishment, *in vivo* fluorescence imaging was performed to measure hepatic expression of the specific shRNA to determine if shRNA activity was sustained in the liver. The rats were sacrificed at week 12 and the liver, myocardium, lung, spleen, and muscle tissues were collected for quantification of shRNA expression in these organs using a confocal laser scanning microscope.

#### 3.5 Animal sacrifice procedure

These procedures are in accordance with guidelines for pain management and humane animal sacrifice. Every 2 weeks, rats were placed with Isoflurane inhalation anesthesia to induce sleep and anesthesia (as preliminarily ascertained). The glass jar was securely covered for 2 min, and next to performed ultrasound measurements of parameters and related drug injections by different groups. After 12 weeks, all experimental animals collected blood before they were intraperitoneally anesthetized with 2% pentobarbital sodium (Syntec, USA) at 100 mg/kg. At the end of the treatment protocols the rats were sacrificed and anaesthetized by CO_2_ inhalation shortly, and collected to liver specimens. Specimens to be used for immunohistochemical staining were fixed in 4% formaldehyde, while the remaining specimens were stored at -80°C prior to RNA and protein extraction.

#### 3.6 Pathological observations

Week 12 was set as the cut-off point for observations. After completion of the ultrasound observations, portal vein puncture was performed for measurement of the portal venous pressure. After blood collection by cardiac puncture, in vivo liver lavage was performed. Rat liver specimens (1 cm in size; n = 5 per group) were taken, fixed in 4% formaldehyde, embedded in paraffin, and cut into slices for hematoxylin and eosin (H&E) staining and for Masson’s staining. All staining results were assessed by two pathologists to determine the stage of hepatic fibrosis in accordance with the METAVIR criteria [14].

#### 3.7 Western blotting for quantifying the expression levels of collagen I, collagen III, MMP1, TIMP1, TGF-β1, and TβR1

An appropriate amount of liver tissue was homogenized, and total liver protein was extracted and quantified using the bicinchoninic acid (BCA) protein assay kit [15]. The sample was subjected to sodium dodecyl sulfate-polyacrylamide gel electrophoresis (SDS-PAGE), transferred to a nitrocellulose membrane, and blocked with 5% nonfat dry milk for 2 h. Subsequently, the primary antibodies (collagen I, collagen III, MMP1, TIMP1, TGF-β1, and TβR1 monoclonal antibodies) were added for overnight incubation at 4°C on a shaker. The membrane was washed three times (5 min each wash) with Tris-buffered saline (TBS) with 0.1%, and the secondary antibody was added for incubation at room temperature for 1.5 h. After incubation, the membrane was washed, and a gel imaging system was used to determine the relative expression level of each target protein in terms of the grayscale ratio of the target protein band to the β-actin (reference) band.

#### 3.8 Statistical methods

SPSS version 19.0 (IBM Corp., USA) was used for statistical analysis of the data. Quantitative data were expressed as means ± standard deviation ($\bar{\chi }$ ± s), and one-way analysis of variance (ANOVA) was used for comparison of quantitative data among multiple groups. P-value < 0.05 was used to indicate significance.

## Results

### 1. Recombinant AAV-TβR1 effectively alleviated collagen deposition in the liver of rats with hepatic fibrosis

To verify the therapeutic effects of recombinant AAV-TβR1 on liver structure, we performed H&E and Masson’s staining on liver tissues taken from the different groups. The H&E and Masson’s staining results indicated normal liver structures in the control group rats. In contrast, staining results for the rats in the model group and in the AAV-TβR1 4W group indicated the occurrence of varying degrees of hepatic fibrosis, which was characterized by a significant increase in collagen fibers; substantial fibrous hyperplasia in the portal region accompanied by inflammatory cell infiltration; broad fibrous septa extending deep into the liver tissue and resulting in the formation of pseudolobules of varying sizes; and the occurrence of steatosis in a portion of the liver cells. For theAAV-TβR1 0W group and the combination therapy group, the degree of hepatic fibrosis in the rats was significantly reduced, which was indicated by a smaller extent of damage to normal liver structures and thin fibrous septa accompanied by unapparent cell degeneration and necrosis. Results of fibrosis staging revealed the occurrence of F4 fibrosis in the majority of rat liver tissues of the model group and of the AAV-TβR1 4W group, and of F3 fibrosis in the majority of rat liver tissues of the AAV-TβR1 0W group and of the combination therapy group (Table 1). Taken together, these results suggest that recombinant AAV-TβR1 can alleviate CCl4-induced hepatic fibrosis in this rat model.

**Table 1:** Staging of hepatic fibrosis in rats of the different groups.

Hepatic fibrosis stage						
Group	Number	F0	F1	F2	F3	F4
Control (A)	10	8	2	0	0	0
Model (B)	10	0	0	0	3	7
0-week therapy (C)	10	0	0	2	6	2
4-week therapy (D)	10	0	0	0	5	5
Combination therapy (E)	10	0	0	3	5	2

### 2. Recombinant AAV-TβR1 effectively reduced the expression levels of collagen I, collagen III, MMP1, TIMP1, TGF-β1, and TβR1 in the liver of rats with hepatic fibrosis

**Fig 3:**
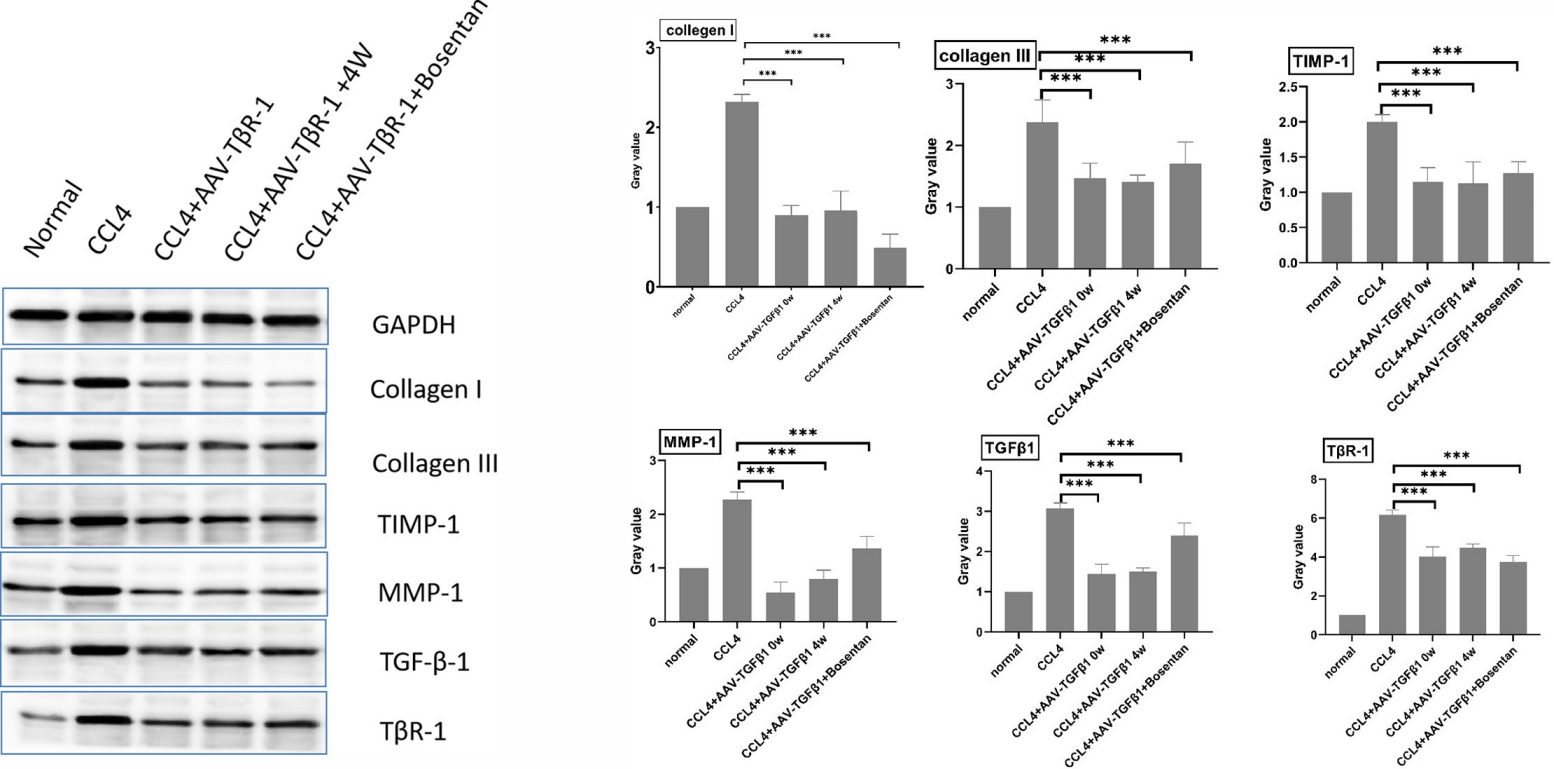
Results of western blotting of the different groups.

Results of western blotting performed on proteins extracted from liver tissues of rats belonging to the different groups indicated that the expression levels of collagen I, collagen III, MMP1, TIMP1, TGF-β1, and TβR1 were significantly higher in the model group than in the control group(Fig 3A and 3B). In both AAV- TβR1 therapy groups and in the combination therapy group, the expression levels of collagen I, collagen III, MMP1, TIMP1, TGF-β1, and TβR1 in the liver of rats with CCl_4_-induced hepatic fibrosis were significantly lower than in the model group. The MMP-1 expression level of the combination therapy group was significantly higher than that of the AAV- TβR1 0W group and of the AAV- TβR1 4W.

### 3. Observation of in vivo viral transfection in rats

**Fig 4:**
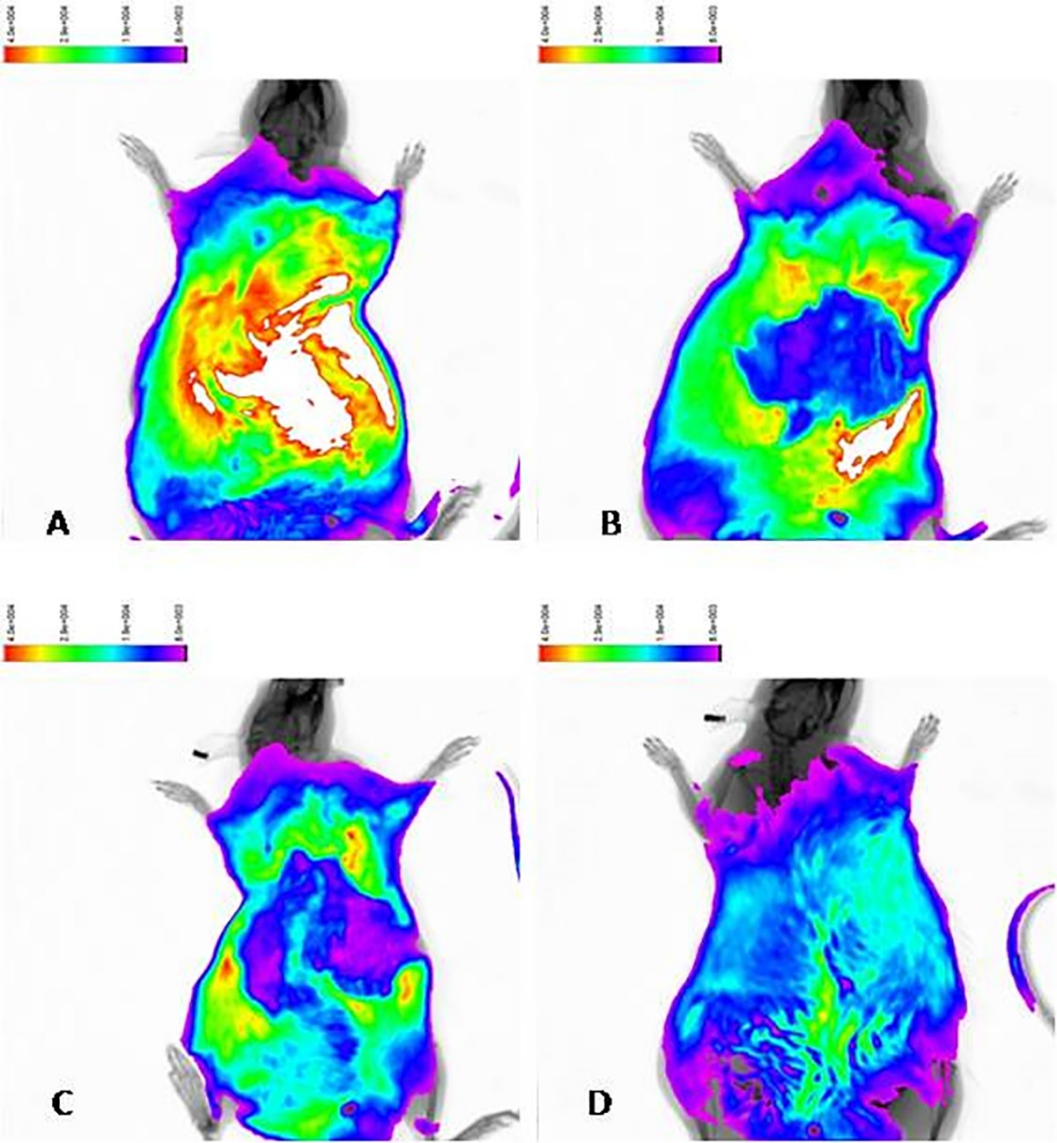
Results of fluorescence imaging performed at (A) week 2, (B) week 6, and (C) week 12 after injections of AAV-TβR1 indicate fluorescence signals of high intensity in the liver. (D) No fluorescence signal was emitted in the control group after injections with saline solution.

Based on the results of *in vivo* fluorescence imaging performed at weeks 2, 4, 6,8, 10, and 12, the control and model groups did not emit significant fluorescence signals, whereas the rats of the AAV- TβR1 therapy groups and of the combination therapy group had fluorescence emissions. In particular, fluorescence intensity was the highest in the liver region, which indicated specific and continuous expression of AAV-TβR1 in the liver (Fig 4). At week 12, measurement of shRNA expression in the liver, myocardium, lung, spleen, and muscle tissues were also performed using a confocal laser scanning microscope. For the control and model groups, no significant fluorescence signal was detected; for the AAV-TβR1 therapy groups and the combination therapy group, fluorescence signals of various intensities were detected in all the organs, with significantly increased fluorescence intensity in the liver.

### 4. Results of ultrasound shear wave elastography

**Fig 5:**
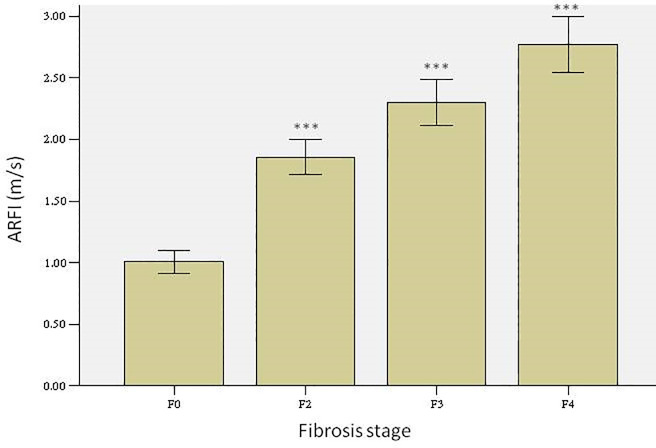
Comparison of the elasticity values of different fibrosis stages.

Using ultrasound shear wave elastography, the liver ultrasound elasticity values of the rats in the different groups were measured, and the elasticity values at the respective fibrosis stages based on the results of pathological examinations were used as standard values for statistical analysis. Results indicated that, based on the model group, the elasticity values corresponding to different stages of hepatic fibrosis were significantly different (Fig 5, p < 0.05), suggesting that elastography can distinguish different degrees of hepatic fibrosis based on liver stiffness, which demonstrates the ability of shear wave elastography to reflect liver stiffness at different stages of hepatic fibrosis. Therefore, this technique can be used to investigate the changes in liver stiffness after AAV-TβR1 therapy and targeted combination therapy. These changes in elasticity values based on liver stiffness indirectly reflect the changes in the degree of hepatic fibrosis and provide a way to monitor the effects therapy against hepatic fibrosis.

**Fig 6:**
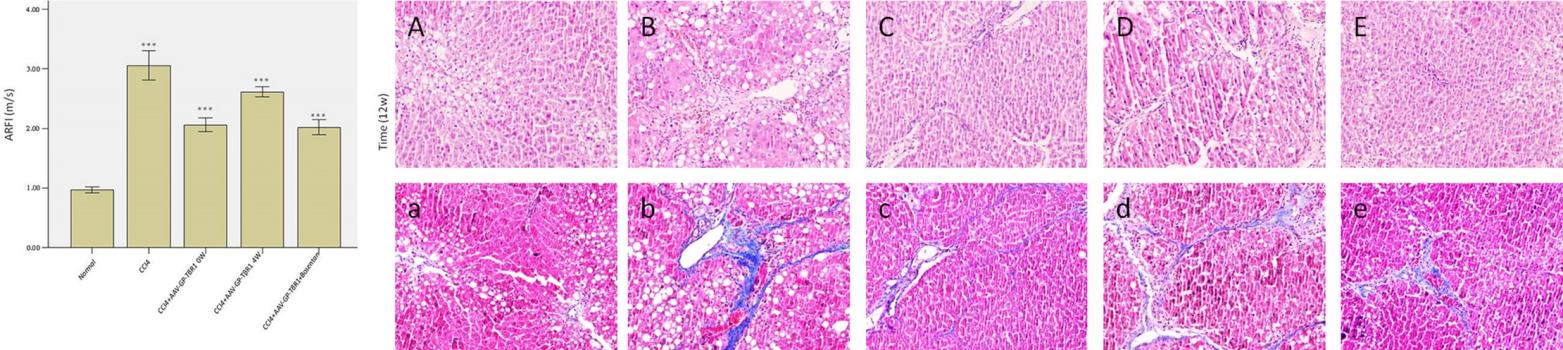
a. Comparison of the elasticity values of the different groups. b. Comparison of the H.E. and Masson’s staining of the different groups(A-E: H.E. staining A:Nomal; B: CCl4; C: CCl4+AAV-GP-TβR1-0W; D: CCl4+AAV-GP-TβR1-4W; E:CCl4+AAV-GP-TβR1 +Bosentan /a-e: Masson’s staining a: Nomal; b:CCl4; c:CCl4+AAV-GP-TβR1-0W;d:CCl4+AAV-GP-TβR1-4W; e:CCl4+AAV-GP-TβR1 +Bosentan).

Results of ultrasound elastography revealed significant differences in the degrees of hepatic fibrosis between the various groups (Fig 6A). The AAV-TβR1 0W group and combination therapy group exhibited significant treatment effects towards hepatic fibrosis in rats, as shown by a significant reduction in liver stiffness compared to the model group (p < 0.01); the pathological results also indicated a decrease in the degree of hepatic fibrosis (Fig 6B). For the AAV -TβR1 4W group, the treatment effects were poorer compared with the effects of the early therapy group (p < 0.05). These results demonstrate that early treatment of hepatic fibrosis is a key link in the retardation of the pathological process of fibrosis, and that effects of treatment can be adequately reflected by ultrasound elastography.

**Fig 7:**
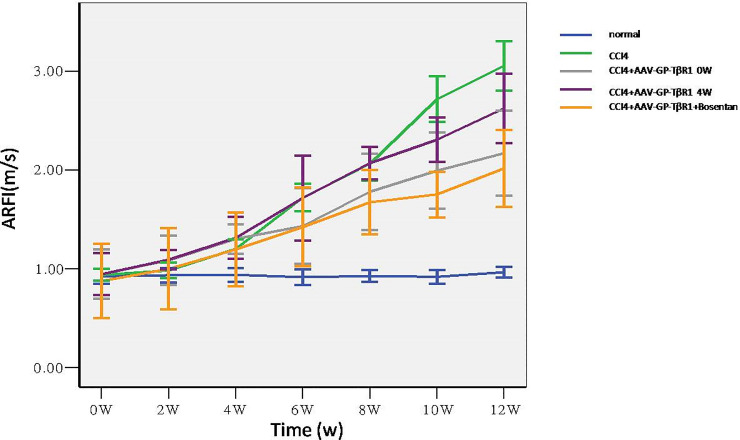
Trend of elasticity values among different groups: Elasticity values of the AAV- TβR1 0W group and of the combination therapy group differed from that of the AAV-TβR1 4W group.

Results of ultrasound elastography performed at weeks 2, 4, 6,8, 10, and 12 indicated that the liver elastic stiffness values of the model group exceeded the values obtained from the control and therapy groups from week 2 onwards, with the differences in elasticity values increasing gradually as the experiment progressed (Fig 7). At week 6 of the experiment, the elasticity values of the AAV- TβR1 0W group and combination therapy group started to differ from the elasticity values of the AAV- TβR1 4W group. However, there was no significant difference between the AAV- TβR1 0W group and the combination therapy group (Fig 7). Taken together, these results indicate that early intervention therapy had an influence on the pathological process of fibrosis.

### 5. Comparison of portal vein diameter and portal venous pressure

**Fig 8:**
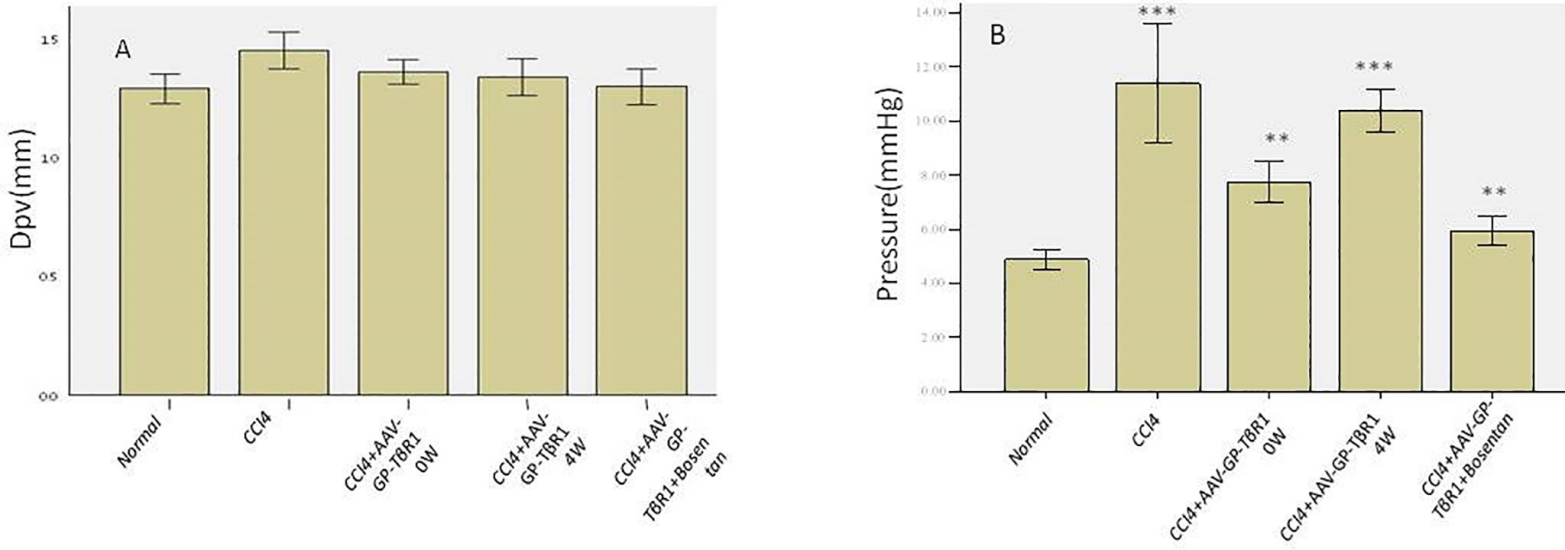
Comparison of portal vein diameter and portal venous pressure among the various groups: (A) portal vein diameter did not differ significantly among the groups. (B) Portal venous pressure values differed significantly between the experimental groups and the control group.

Although there were no significant differences in portal vein diameter among the various groups, the portal vein diameter of the model group was slightly larger than the portal vein diameters of the other groups (Fig 8A). Measurements of portal venous pressure revealed significant differences between the experimental groups and the control group (p < 0.01), which shows that anti-fibrosis therapy can significantly reduce portal venous pressure, with the effects of early and combination therapy being significantly superior to that of intervention therapy at Week 4 (p < 0.05) (Fig 8B). Positive correlations were identified between portal venous pressure and the degree of fibrosis, and between liver elastic stiffness and portal venous pressure. Therefore, in addition to reducing the degree of fibrosis, early combination therapy also effectively reduced portal venous pressure.

### 6. Comparison of abdominal aortic elasticity parameter

**Fig 9:**
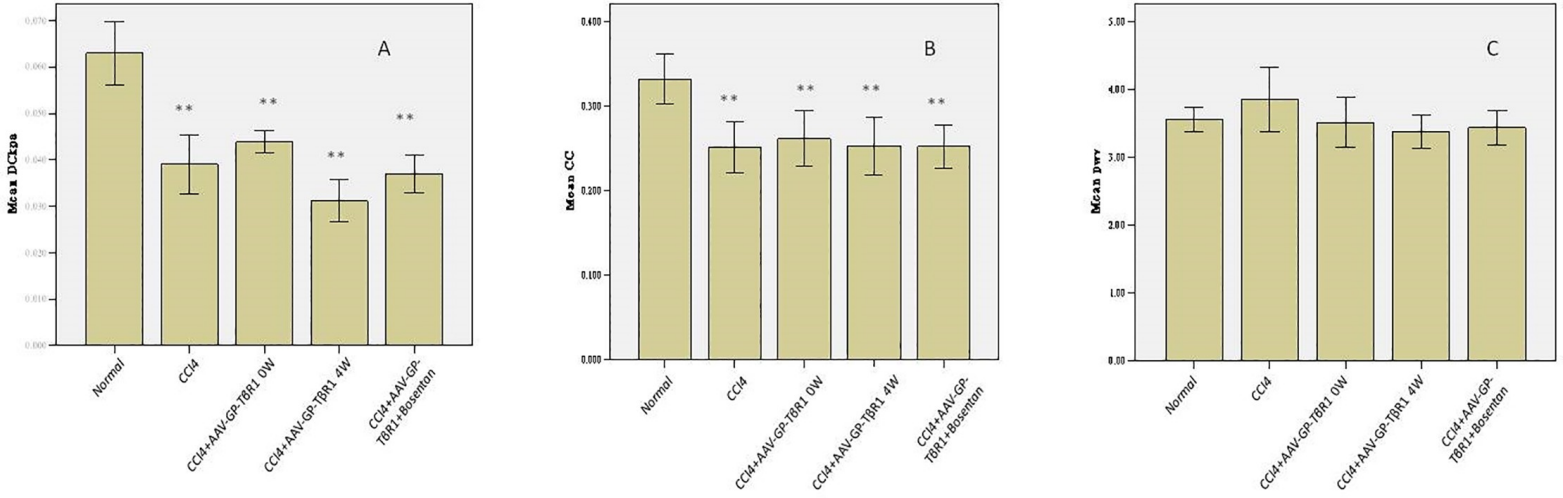
Comparison of abdominal aortic elasticity parameter values: (A)/(B) differences existed between the experimental groups and the control group, but differences among the various experimental groups were not statistically significant. (C) There were no significant differences in PWV between the experimental groups and the control group.

The abdominal aortic elasticity parameter differed significantly between the experimental groups and the control group (p < 0.05); however, the differences among the experimental groups were not statistically significant (Fig 9A and 9B). There were no significant differences in the abdominal aortic PWV between the experimental groups and the control group (Fig 9C). These results indicate that changes occurred in the abdominal aortic elasticity parameters of rats with hepatic damage.

## Discussion

Gene therapy has great promise as a possible treatment method for hepatic fibrosis. Previous studies have identified the TGF-β1 receptor in the TGF-β1 signaling pathway as a crucial point of action since the blockage or silencing of this receptor affects a series of downstream factors, ultimately leading to decreased synthesis of the ECM [3,4]. However, the development of methods for the safe, highly effective and targeted delivery of genes into specific organs and tissues for expression remains as a primary hurdle for gene therapy. In the present study, we designed a specific shRNA lentiviral vector for targeted therapy against hepatic fibrosis. The experimental results indicated a high transfection rate and effective downregulation of collagen I, collagen III, MMP1, and TIMP1 in rat liver. These results demonstrate that the silencing of TβR1 can effectively prevent ECM formation and achieve anti-fibrotic effects. In addition, pathological observations using a rat model for hepatic fibrosis showed a significantly lesser degree of fibrosis in the intervention groups than in the untreated model group. Additionally, the degree of fibrosis in the 0W intervention group was lower than that in the 4W intervention group, which indicates that early treatment can effectively alleviate the progression of hepatic fibrosis.

Ultrasound elastography, which can reflect the texture and stiffness of organs, has been useful in the examination of organs such as the thyroid gland, mammary gland, and liver. Previous studies have reported that ultrasound elastography is able to distinguish different degrees of fibrosis in chronic liver disease and that the results of ultrasound elastography are also well-correlated with the pathological results of biopsies [7–10]. Therefore, ultrasound elastography can be used as a non-invasive method for determining the degree of fibrosis. As such, it can also be potentially used to observe the effects of anti-fibrosis therapy. In this study, we utilized the ARFI technique to perform ultrasound elastography on a rat model of hepatic fibrosis at different time points to observe the influence of various therapies. Our results indicated that the ARFI values of the experimental groups and of the control group did not differ significantly during 0 to 4 weeks. From week 6 onwards, the ARFI value of the model group became significantly higher than the ARFI values of the therapy groups. Results of pathological examination conducted at week 12 also indicated lesser degree of hepatic fibrosis in the therapy groups than in the model group. These results demonstrate that shRNA therapy against TβR1 as well as its combination with bosentan significantly alleviate the pathological process of hepatic fibrosis, and the associated pathological changes are adequately reflected by the ARFI values. Based on the pathological results as standard for analysis of the ARFI values of rats with different degrees of hepatic fibrosis, we found that the ARFI value of the F2 group was lower than that of the F3 group; the ARFI value of the F3 group was lower than that of the F4 group; and the ARFI values among the various groups were significantly different. These results are similar to those of other studies, indicating that ultrasound elastography can be used to identify different pathological stages of fibrosis [16,17].

In the present study, the influence of the timing of treatment on the pathological process of hepatic fibrosis was also investigated. Our results showed that the administration of targeted combination therapy immediately after the establishment of the hepatic fibrosis rat model led to a significantly lower ARFI value compared to the administration of targeted therapy from week 4 onwards. Results of the pathological examination performed at week 12 also indicated that the 0W group had a lesser degree of fibrosis than the 4W group. Therefore, early anti-fibrosis therapy plays a crucial role in the retardation of the fibrotic process. Since ultrasound elastography is able to reflect the differences between the effects of treatment timing, it can be used for monitoring the effects of anti-fibrosis therapy.

One of the hemodynamic changes typically induced by hepatic fibrosis is high portal venous pressure. In existing literature, there is no consensus on whether portal vein diameter is reflective of portal venous pressure [18]. We performed ultrasound examinations for the measurement of portal vein diameter at different observation time points, and results indicated that portal vein diameter did not differ between the experimental groups and the control group. However, the portal venous pressure measurements performed at week 12 showed that the portal venous pressure values obtained from the experimental groups were significantly higher than the value obtained from the control group. Additionally, the portal venous pressure of rats in the combination therapy group was significantly higher than that of the model group and of the 4W therapy group. Therefore, our results indicate that portal vein diameter does not provide a timely reflection of the portal venous pressure. Although variations in portal vein diameter as reported by previous literature were also observed during the fibrotic process in this study, the changes in portal venous pressure were marginally higher than the changes in portal vein diameter as fibrosis progressed [18,19]. Therefore, the portal vein diameter is not an ideal indicator for monitoring changes in portal venous pressure and is unable to reflect the changes following anti-fibrosis therapy.

In summary, TGF-β1 signaling pathway-targeted TβR1 silencing, especially when administered early in the progression of the disease, can effectively retard the pathological process and reduce the degree of hepatic fibrosis. The ARFI technique of ultrasound elastography can adequately reflect pathological changes induced by anti-fibrosis therapy and is beneficial in monitoring the effects of anti-fibrosis therapy through time. Therefore, it is a potentially useful non-invasive technique for observing the effects of treatment in hepatic fibrosis patients, and further research on this method is of practical significance to the reduction of the use of liver biopsy in future clinical practice. However, further studies are required to investigate the application of this method in determining the early effects of anti-fibrosis therapy so as to enhance the sensitivity and specificity of this technique.

## Supporting information

S1 Checklist PLOS ONE clinical studies checklist. (DOCX)
